# Use of mid-upper arm circumference for determining overweight and overfatness in children and adolescents

**DOI:** 10.1136/archdischild-2013-305137

**Published:** 2014-06-02

**Authors:** E Craig, R Bland, J Ndirangu, J J Reilly

**Affiliations:** 1 Section of Human Nutrition, University of Glasgow, Yorkhill Hospitals, Glasgow, UK; 2 Africa Centre for Health and Population Studies, University of KwaZulu-Natal, Mtubatuba, KwaZulu-Natal, South Africa; 3 Royal Hospital for Sick Children, University of Glasgow, Glasgow, UK; 4 University of Strathclyde, School of Psychological Sciences & Health, Glasgow, UK

## Abstract

**Objective:**

To assess the use of mid-upper arm circumference (MUAC) for identification of overweight and overfatness in rural South African children and adolescents.

**Methods:**

Anthropometric data (weight, height, MUAC and % body fat) from a cross-sectional sample of 978 black South African 5–14-year-olds were analysed. Receiver operating characteristic (ROC) curve analysis determined the validity of MUAC as a proxy for determining overweight and overfatness.

**Findings:**

Area under the curve (AUC) results were generally high. Boys and girls aged 10–14 years had ROC-AUC for overfatness classed as ‘excellent’, 0.97 and 0.98 respectively. Cut-points in the MUAC distribution which optimised the ROC-AUC for identification of overfatness and obesity were determined for boys and girls aged 5–9 and 10–14 years, and had high sensitivity and specificity.

**Conclusions:**

MUAC may have potential for clinical and surveillance applications as an accurate yet simple and widely available indicator of overweight and overfatness in children and adolescents in resource-poor settings.

**What is already known on this topic**

- Mid-upper arm circumference (MUAC) is widely used as an indicator of severe and moderately acute undernutrition, with simple guidelines available for children aged up to 5 years.
- It is not known if MUAC is helpful as an indicator of overweight or overfatness in older children and adolescents.

**What this study adds**

- MUAC can identify overweight and overfatness with reasonable accuracy in school-age children and adolescents.
- MUAC may have potential as a clinical and surveillance indicator of obesity in children and adolescents.

## Introduction

Childhood obesity has become pandemic, and has short-term and long-term adverse impacts on health.^1^ Body mass index (BMI)-for-age is recommended internationally as the optimal, simple measure of obesity for public health surveillance and clinical applications in children and adolescents.^2^ However, there are several practical barriers associated with this method particularly in resource-poor settings where equipment and training are limited. If a simpler, more practical alternative to BMI was available this may increase clinical assessments of obesity, and enhance obesity surveillance in resource-poor settings where data on the prevalence of overweight and obesity among older children and adolescents are sparse.^3^

Mid-upper arm circumference (MUAC) is an easy, quick and inexpensive field measure most commonly used for identification of severe acute undernutrition in young children (6–60 months of age) in resource-limited settings. There is a dearth of research on the potential for MUAC as a screening tool for obesity, or its use in older children and adolescents.

The present study aimed to determine the accuracy of MUAC for the assessment of overweight (as defined on the basis of BMI-for-age) and overfatness (as defined by bioimpedance estimates of body fatness) in children and adolescents.

## Methods

### Study setting, participants and anthropometric measures

Study participants were black Zulu children and adolescents from school grades 1, 5 and 9 (approximate ages 7, 11 and 15 years) living within the Africa Centre Demographic Surveillance Area (DSA) in KwaZulu–Natal, South Africa, who were enrolled in a larger cross-sectional study which aimed to determine prevalence of overweight and obesity in this area.^4^ All measurements (height, weight, MUAC and % body fat estimates) were carried out by trained field workers, and standard operating procedures were in adherence with WHO standards. Overweight (including obesity) was defined in the present study using the WHO 2007 BMI-for-age reference where overweight is classified as having a z score >+1SD.^2^ Overfatness was defined using McCarthy 2006 body fat reference curves for children and adolescents based on bioimpedance (TANITA SC240MA). Cut-offs for excess fatness were age and sex-specific, and defined as the 85th percentile of the McCarthy reference.^5^

### Statistical analysis

All statistical analysis was carried out using STATA V.11.0.

Receiver operating characteristic (ROC) analysis was used to test the ability of MUAC to determine those children and adolescents identified as overweight by BMI-for-age and overfat by bioelectrical impedance. For the analysis, participants were divided into sex-specific (male and female) and age-specific child (5–9 years) and adolescent (10–14 years) groups, these age groups were chosen based on previously published MUAC guidelines.^6^ The categories used to summarise accuracy in ROC analysis were as follows: 0.9–1 Excellent, 0.8–0.9 Good, 0.7–0.8 Fair, 0.6–0.7 Poor, 0.5–0.6 Fail.

The area under the curve (AUC) is a measure of accuracy and is indexed from 0 to 1 where 1 indicates a perfect test and ≤0.5 a worthless test.

A probability curve was plotted to determine the probability of being overweight or overfat at each MUAC reading. The point at which the probability curve showed a marked increase was selected as a possible indicator. Based on the results of the ROC curve and the probability graph, two potential optimal operating points (OOP) were determined, and their accompanying data with regards to probability, sensitivity, specificity and classification accuracy was calculated.

Two potential cut points were chosen for each age-specific and gender-specific group, these potentially OOPs aimed to maximise sensitivity and specificity, while also taking into account results of the ROC curve, probability curve and level of classification accuracy, with the intention that future studies would be able to verify which of the two cut-points is most suitable, and how generalisable they might be to different populations.

## Results

### Characteristics of study participants

A total of 978 participants were included in this analysis (481 children aged 5–9 years (235 and 246 females and males, respectively) and 497 aged 10–14 years (269 and 228 females and males, respectively) (see table 1).

**Table 1:** Characteristics of study participants

Age group (years)	Sex	n	Age (years) mean (SD)	Body fat % mean (SD)	BMI z score mean (SD)
5–9	Female	235	7.2 (0.8)	19.5 (5.2)	−0.02 (0.99)
	Male	248	7.2 (0.8)	17.2 (3.1)	−0.16 (0.97)
10–14	Female	269	11.6 (1.2)	21.3 (6.0)	−0.01 (1.01)
	Male	228	11.8 (1.01)	13.4 (5.2)	−0.31 (1.02)

#### Ability of MUAC to classify overfatness correctly

AUC results from the ROC curve were ‘excellent’ for girls and boys 10–14 years of age (0.97 and 0.98, respectively); for girls and boys aged 5–9 years classification accuracy was ‘good’ (0.88) for girls and ‘poor’ (0.66) for boys.^5^

In general, sensitivity and specificity were relatively high for the cut-points chosen for all age and gender groups (table 2). The exceptions were in boys aged 5–9 years where sensitivity was around 25%, and girls aged 10–14 years where the specificity was around 69–77% depending on the cut-points chosen. The percentage of individuals correctly classified using the potentially optimal cut points from the ROC analysis ranged from 72% to 94% (table 2).

**Table 2:** Determining optimal operating point (OOP) for MUAC versus overfat

MUAC (cm)	Predicted probability	Sensitivity (%)	Specificity (%)	Correctly classified (%)	Positive likelihood ratio (LR+)	Negative likelihood ratio (LR−)
Girls 5–9						
19.2	0.32	63.8	90.4	85.1	6.67	0.4
19.45	0.37	57.5	94.7	87.2	10.8	0.45
Boys 5–9						
18.4	0.21	27.9	85.7	75.6	1.95	0.84
18.65	0.22	25.6	90.6	79.3	2.73	0.82
Girls 10–14						
22.0	0.02	100	69	72.5	3.23	0.00
22.6	0.03	100	77.4	79.9	4.43	0.00
Boys 10–14						
23.2	0.06	92.3	94.0	93.9	15.27	0.08
23.6	0.08	92.3	94.9	94.7	18.04	0.08

MUAC, mid-upper arm circumference.

Based on the ROC analysis and probability curve, the following optimal MUAC cut-points to identify overfatness were identified: 5–9 years 19.2 cm/19.5 cm and 18.4 cm/18.7 cm for girls and boys, respectively; at ages 10–14 years, 22.0 cm/22.6 cm and 23.2/23.6 cm for girls and boys, respectively.

### Ability of MUAC to classify overweight (including obesity) status defined by WHO 2007 BMI-for-age reference

The ROC–AUC for MUAC versus overweight was ‘excellent’ for girls and boys in both age groups (0.96 and 0.90 in girls and boys in the younger age group; 0.94 and 0.97 in girls and boys in the older age group).^2^ Sensitivity and specificity were generally high for the optimal age-specific and gender-specific cut-points (76–97%) (table 3). The percentage of individuals correctly classified using the proposed cut-points ranged from 80% to 92% across age and gender groups (table 3).

**Table 3:** Determining optimal operating point (OOP) for MUAC versus overweight/obesity

MUAC (cm)	Predicted probability	Sensitivity (%)	Specificity (%)	Correctly classified (%)	Positive likelihood ratio (LR+)	Negative likelihood ratio (LR−)
Girls 5–9						
18.3	0.07	97.1	79.1	81.7	4.65	0.037
18.85	0.14	94.1	88.1	88.9	7.88	0.066
Boys 5–9						
18.4	0.14	68.0	89.2	87.10	6.32	0.3586
18.6	0.18	64.0	93.3	90.3	9.51	0.3860
Girls 10–14						
22.45	0.08	92.9	78.0	80.3	4.22	0.09
22.8	0.12	88.1	81.1	82.2	4.65	0.15
Boys 10–14						
22.15	0.07	95.2	89.9	90.4	9.39	0.05
23.15	0.17	76.2	76.2	92.5	13.14	0.25

MUAC, mid-upper arm circumference.

Based on ROC analysis and probability curve, the following potentially optimal MUAC cut-offs to identify overweight are proposed: ages 5–9 years, 18.3 cm/18.9 cm and 18.4 cm/18.6 cm for girls and boys, respectively; at ages 10–14 years, 22.5 cm/22.8 cm and 22.2 cm/23.2 cm for girls and boys, respectively.

## Discussion

### Main findings

In the present study, the classification accuracy of MUAC was high for overweight (defined by BMI), and for overfatness (defined by bioelectrical impedance), although accuracy was higher for BMI (a weight-based measure) than for body fatness. Sensitivity, specificity and AUC were generally high relative to similar studies of the classification accuracy of BMI and waist circumference in children and adolescents as summarised in two recent systematic reviews^7^. 8 Further research would be necessary to determine the reason for poor accuracy in boys aged 5–9 years.

The present study provides a ‘proof of concept’ of the potential for MUAC to define overnutrition. The present study should not provide the basis of a recommendation to use MUAC to define overnutrition, and the cut-points identified as potentially optimal should only be used with caution. Ideally, the potentially optimal MUAC cut-points from the present study should be tested in an independent sample in order to provide confidence in the ability of MUAC to detect overweight and overfatness and analysis of alternative age groupings or even single ages may also be necessary. Adjustments may also be necessary for stunting status and pubertal development given that these factors have been found to impact BMI and body fat status and, therefore, may impact interpretation of results. Future research should also investigate the functional outcomes of MUAC, that is, the extent to which a high MUAC predicts the development of the comorbidities of obesity, such as diabetes and cardiovascular disease.

### Implications

Our findings suggest that MUAC has potential as a proxy measure for overweight and overfatness in children and adolescents in rural South Africa and probably other resource-poor settings. Measurement of MUAC is a simple, practical proxy for undernutrition,^9^ but the present study suggests that it might have future potential for public health surveillance of obesity, and as a screening tool to identify children or adolescents who might benefit from further assessment and/or clinical management.

### Strengths and limitations

Few studies have measured MUAC in large samples; the majority of research, to date, has focussed on children under 5 years of age and predominantly on the use of MUAC for the assessment of undernutrition. One limitation of the present study is that the optimal cut-offs in the MUAC distribution were not cross-validated in an independent sample which would be necessary to establish the value of MUAC for the assessment of overweight and overfatness, The present study used the widely accepted and recommended BMI-for-age cut-offs to define overweight (including obesity), but for overfatness, the McCarthy body fat reference curve was used.^5^ The applicability of the McCarthy *et al* definitions of overfatness, based on a European population, to other populations is unclear at present, but we note that these definitions use what would generally be accepted to be very high levels of body fatness in all populations. An alternative approach would have been to use a criterion method of body fatness measurement (a 3 or 4 component model) to define overfatness, but the criterion methods are not practical for use in large samples in resource-limited settings.

In summary, the present study provides novel evidence that MUAC has the potential to be used in the screening of overnutrition, and calls for further studies to investigate this concept in other populations. Future research may also seek to determine the effect puberty and different obesity phenotypes have on MUAC, as well as assessing functional outcomes of a high MUAC.

## References

[1] Reilly JJ Kelly J Long-term impact of overweight and obesity in childhood and adolescence on morbidity and premature mortality in adulthood: systematic review. Int J Obes (Lond) 2011;35:891–8. 20975725 10.1038/ijo.2010.222

[2] WHO. WHO Child Growth Standards. 2007 [cited 2013 24 April]; http://www.who.int/childgrowth/en/

[3] Prentice AM The emerging epidemic of obesity in developing countries. Int J Epidemiol 2006;35:93–9. 16326822 10.1093/ije/dyi272

[4] Craig E Reilly J Bland R Body fatness or anthropometry for assessment of unhealthy weight status? Comparison between methods in South African children and adolescents. Public Health Nutr 2012;16:1–9 . 10.1017/S1368980012004338 PMC10271513 23034177

[5] McCarthy HD Cole TJ Fry T Body fat reference curves for children. Int J Obes (Lond) 2006;30:598–602. 16570089 10.1038/sj.ijo.0803232

[6] WHO. Guidelines for an intergrated approach to the nutritional care of HIV-infected children (6 months-14 years). 2009. 23785741

[7] Reilly JJ Wilson ML Summerbell CD Obesity: diagnosis, prevention, and treatment; evidence based answers to common questions. Arch Dis Child 2002;86:392–4. 12023163 10.1136/adc.86.6.392 PMC1762983

[8] Reilly JJ Kelly J Wilson DC Accuracy of simple clinical and epidemiological definitions of childhood obesity: systematic review and evidence appraisal. Obes Rev 2010;11:645 55. 20059704 10.1111/j.1467-789X.2009.00709.x

[9] Goossens S Bekele Y Yun O Mid-upper arm circumference based nutrition programming: evidence for a new approach in regions with high burden of acute malnutrition. PLoS ONE 2012;7:e49320. 23189140 10.1371/journal.pone.0049320 PMC3506602

